# Yeasts as Biopharmaceutical Production Platforms

**DOI:** 10.3389/ffunb.2021.733492

**Published:** 2021-09-22

**Authors:** Natalja Kulagina, Sébastien Besseau, Charlotte Godon, Gustavo H. Goldman, Nicolas Papon, Vincent Courdavault

**Affiliations:** ^1^Université de Tours, EA2106 Biomolécules et Biotechnologies Végétales, Tours, France; ^2^Université d'Angers, EA3142 Groupe d'Etude des Interactions Hôte-Pathogène, Angers, France; ^3^Departamento de Ciências Farmacêuticas, Faculdade de Ciências Farmacêuticas de Ribeirão Preto, Universidade de São Paulo, Ribeirão Preto, Brazil

**Keywords:** yeast, biopharmaceuticals, natural products, heterologous production, metabolic engineering

## Introduction

Biopharmaceuticals and medicinal natural products (NPs) embrace a vast and continuously expanding range of applications in human medicine, which requires high-scale production to meet clinical demands and prevent natural source-related restraints. The development of genetic engineering tools and genome sequencing enabled the emergence of heterologous production as an alternative supply strategy. One of the pioneering examples is *Escherichia coli*-produced insulin, which was Food and Drug Administration (FDA)-approved and commercialized in 1982, followed by the *E. coli*-produced growth hormone (GH) in 1985 (Wang et al., 2017). Currently, apart from gonadotropic hormones, which are produced only in mammalian cells given their specific glycosylation (Orvieto and Seifer, 2016), most of the other commercialized hormones are synthesized by recombinant *Saccharomyces cerevisiae* (insulin, glucagon, GH) or *E.coli* [insulin, GH, glucagon, calcitonin, teriparatide (Walsh, 2018)]. Over the past decades, the constantly broadening biopharmaceutical industry exhibited the blossom of vaccines, therapeutic monoclonal antibodies (mAbs), recombinant enzymes, cytokines and blood-related proteins, most of which are produced in mammalian cells (predominantly Chinese hamster ovary cells, CHO) (Walsh, 2010, 2018), a time-consuming, laborious and expensive process. Microbe expression system-derived recombinant proteins, on the other hand, face several limitations, such as post-translational modifications (PTMs), potential immunogenicity, poor stability and short serum half-life. One of the preferred heterologous expression systems are generally recognized as safe (GRAS) yeasts (e.g. *S. cerevisiae, Pichia pastoris, Yarrowia lipolytica, Hansenula polymorpha)*, which are robust, easy to genetically manipulate, cost-effective, and unlike *E. coli* possess native PTM machinery and lack endotoxins (Demain and Vaishnav, 2009; Martínez et al., 2012). Still, yeast glycosylation differs from human N- and O- glycosylation, and to circumvent this limitation a major effort was directed toward yeast glycoengineering. In addition, yeast secretory machinery and endogenous protein degradation were targeted to enhance recombinant protein-folding, secretion and stability (Laukens et al., 2015; Huang et al., 2018). On the other hand, whole yeast-based vaccines (WYVs) emerged as an appealing approach to combat infectious diseases and cancers (Roohvand et al., 2017), further placing yeast in the spotlight. In parallel, the industrial-scale heterologous production of mammal- and plant-derived medicinal NPs was achieved for progesterone (Duport et al., 1998), hydrocortisone (Szczebara et al., 2003) and artemisinic acid (a precursor of antimalarial artemisinin, Paddon et al., 2013), via the complete pathway reconstitution in engineered *S. cerevisiae*. This strategy was applied to other plant-derived active compounds, such as resveratrol (800 mg/L, Li et al., 2016), strictosidine (0.5 mg/L, Brown et al., 2015), thebaine (0.006 mg/L, Galanie et al., 2015), ginsenoside Rh2 (300 mg/L, Zhuang et al., 2017) and noscapine (2.2 mg/L, Li et al., 2018). However, obtaining industry-compatible yields remains challenging due to plant pathway complexity and subcellular architecture, as well as heterologous enzyme poor activity and limiting native metabolism. In this opinion article, we will overview the main classes of recombinant biopharmaceuticals currently commercialized, and highlight the recent progress in the construction and optimization of yeast cell factories for the production of biopharmaceuticals and medicinal NPs.

## Recombinant Therapeutic Proteins

### Vaccines

Since their first massive application in the 1960s, vaccines have considerably improved public health and remarkably reduced child mortality (Pollard and Bijker, 2021). However, besides the time inefficiency of conventional vaccine production, which is particularly problematic concerning the newly emerging diseases, mass cultivation of pathogens displays a high risk of pathogenicity while live attenuated pathogen-based vaccines are incompatible with immunocompromised individuals (Kumar and Kumar, 2019). Thus, protein-based vaccines, virus-like particle (VLP)-based vaccines, and more recently peptide-, viral vector- and nucleic acid-based vaccines have been explored to expand and facilitate vaccine design and production (Kim and Kim, 2017; Ho et al., 2021; Pollard and Bijker, 2021). Subunit-based and VLP-based vaccines employ recombinant strategies and heterologous production in microbial cell factories, and particularly yeast platforms (Mohsen et al., 2017; Roohvand et al., 2017). For instance, recombinant vaccines produced by yeasts have been approved and employed since 1980s, notably the vaccines against hepatitis B virus (HBV) from 1986, and combination vaccines against several pathogens from 1996 (Table 1). WYV strategy, on the other hand, was inspired by adjuvant, anti-cancer and immunomodulatory properties of yeast cell wall β-glucans, which, combined with the ease of yeast genetic manipulation, gave an idea to recombinant WYVs expressing pathogen- or tumor-specific antigens on the cell surface (Roohvand et al., 2017). In *S. cerevisiae*, antigen anchoring to the cell surface is achieved via the fusion with cell wall proteins, such as glycosylphosphatidylinositol (GPI) and protein with internal repeats (PIR) proteins. PIR proteins are directly linked to the cell wall β-glucans while GPI proteins require specific GPI anchors (Ecker et al., 2006; Pittet and Conzelmann, 2006). While the commercialized GPI α-agglutinin system is the most widely used, other GPI systems have been developing to improve cell surface display by assessing different GPI anchors (Yang et al., 2019). Currently, several heat-killed WYVs for oral administration are under clinical or preclinical investigation, targeting various cancers, such as melanoma, papilloma, leukemia and carcinoma (Roohvand et al., 2017; Kumar and Kumar, 2019), and viruses, including influenza H7N9 (Lei et al., 2020) and COVID-19 (Gao et al., 2021).

**Table 1:** FDA-approved biopharmaceuticals produced in yeast cells (adapted from Walsh, 2018).

**Biopharmaceutical**	**Expression system**	**First product**	**Target**
**Hormones**			
Insulin (rh)	*S. cerevisiae*	1991 (Novolin 1991–2010)	Diabetes mellitus
Glucagon (rh)	*S. cerevisiae*	1999 (Glucagen)	Hypoglycemia
Somatropin (rhGH)	*S. cerevisiae*	2006 (Valtropin 2006–2012)	Growth failure
Insulin glargine (r)	*P. pastoris*	2018 (Semglee)	Diabetes mellitus
**Vaccines**			
HBsAg (r)	*S. cerevisiae*	1986 (Recombivax)	Hepatitis B
Combination vaccine (r)	*S. cerevisiae*	1996 (Tritanrix-hepB 1996–2014)	Multiple
HPV capsid proteins (r)	*S. cerevisiae*	2006 (Gardasil)	Human papillomavirus (HPX, 4 types)
VLP (partial surface proteins from *P. falciparum* and HBV*)* (r)	*S. cerevisiae*	2015 (Mosquirix)	*P. falciparum*-caused Malaria; Hepatitis B
Combination vaccine (r)	*H. polymorpha*	2013 (Hexacima)	Multiple
HBsAg (r)	*H. polymorpha*	2017 (HEPLISAV-B)	Hepatitis B
**Blood-related**			
Anticoagulants			
Hirudin (desirudin) (r)	*S. cerevisiae*	1997 (Revasc 1997–2014)	Venous thrombosis, thrombocytopenia
*Clotting factors*			
Plasma kallikrein inhibitor (ecallantide) (rh)	*P. pastoris*	2009 (Kalbitor)	Hereditary angioedema
Factor XIII A-subunit (catridecog) (rh)	*S. cerevisiae*	2012 (Novothirteen)	Congenital factor XIII A-subunit deficiency
Proteolytics			
Truncated plasmin (ocriplasmin) (rh)	*P. pastoris*	2012 (Jetrea)	Vitreomacular adhesion/traction
**Cytokines**			
*Colony-stimulating factors* GM-CSF (sargamostim) (rh)	*S. cerevisiae*	1991 (Leukine)	Neutropenia
**Enzymes**			
Urate oxidase (rasburicase) (r)	*S. cerevisiae*	2001 (Fasturtec)	Hyperuricemia

*r, recombinant; rh, recombinant human*.

### Monoclonal Antibodies

In parallel, therapeutic monoclonal antibodies (mAbs) and targeted immunotherapy emerged as an extensively growing class of medication aimed to treat various human pathologies, such as cancers, cardiovascular, infectious, inflammatory and autoimmune diseases (Kaplon and Reichert, 2019). Following the first murine mAb approved and commercialized in 1986 to prevent kidney transplant rejection, more than 100 mAbs were developed (Walsh, 2018). The success of this approach relies on mAb target specificity mediated by antigen binding domain and its variable regions, and crystallizable fragment domain engaged in mAb function, recycling and higher serum half-life (Buss et al., 2012). In addition, mAb immunogenicity was considerably limited with the development of chimeric (65% human, e.g., neuroblastoma-treating dinutuximab beta in 2017), humanized (95% human, e.g., breast and gastric cancer-treating trastuzumab in 2018) and human (e.g., Merkel cell carcinoma-treating avelumab in 2017) mAbs (Buss et al., 2012; Walsh, 2018). However, the specific glycosylation required for the immune response activation, as well as protein-folding issues, have been restricting mAbs production to mammalian cells (Jung and Kim, 2017). Indeed, yeast *N* glycosylation patterns are of a high mannose type, which is immunogenic and reduces recombinant protein serum half-life in mammals. To overcome this limitation, yeast glycoengineering was engaged to prevent hypermannosylation and humanize glycosylation machinery. For instance, the deletion of *OCH1* gene encoding the mannosyltransferase involved in the outer chain elongation of *N*-linked oligosaccharides, and the expression of heterologous α-1,2-mannosidase resulted in the production of human-mannose type glycans, while the introduction of mammal glycosyltransferases downstream enabled the production of more complex human-type glycans (reviewed in Laukens et al., 2015). Meanwhile, *P. pastoris* emerged as a more attractive expression host given its less extensive hypermannosylation compared to *S. cerevisiae*. Subsequently, several studies demonstrated glycoengineered *P. pastoris* as a platform for the production of humanized mAbs (Bobrowicz et al., 2004; Li et al., 2006; Potgieter et al., 2009).

### Blood Factors and Blood-Related Proteins

The majority of blood factors (e.g., clotting factor VIII; coagulation factors VIIa, IX, Xa, IIa, thrombolytic reteplase) are produced in mammalian cells, except several examples of yeast platforms, such as clotting fibrin stabilizing factor XIIIa expressed in *S. cerevisiae*, which treats congenital factor XIIIa deficiency and is commercialized since 2012, as well as *P. pastoris*-synthesized clotting plasma kallikrein inhibitor and proteolytic recombinant plasmin approved in 2009 and 2012, respectively (Martínez et al., 2012; Korte, 2014; Walsh, 2018) (Table 1). Another anticoagulant is *S. cerevisiae*-produced hirudin from *Hirudo medicinalis* approved in 1997, which was discontinued in 2012, probably due to the risk of overdosing, only manageable by hemofiltration, and several cases of anaphylaxis (Greinacher et al., 2003; Cardenas and Deitcher, 2005; Petros, 2008; Walsh, 2018). Alternative anticoagulants have been explored, such as human antithrombin III that lyses thrombin and factor Xa upon blood coagulation cascade, and is manufactured in the milk of transgenic goats since 2006. More recently, it was reported to be expressed in *S. cerevisiae* yielding 312 mg/L in fed-batch fermentation (Mallu et al., 2016). Recombinant plasma albumin, on the other hand, has been produced in multiple expression systems, including *S. cerevisiae* and *P. pastoris*, which showed the most promising titer of above 3 g/L and 10 g/L in fed-batch cultivation, respectively (Chen et al., 2013). In parallel, the development of blood substitutes for transfusions, notably recombinant hemoglobin (Hb), has been investigated for decades. Several studies displayed promising results in *S. cerevisiae* targeting the native heme biosynthesis by overexpressing *HEM* genes (particularly *HEM3*), and the optimization of recombinant Hb subunit α and β expression via 2:1 gene copy ratio (Liu et al., 2014; Martínez et al., 2015). In addition, the deletion of *HAP1* gene that encodes heme-activated transcription factor, known to initiate the transcription of respiration-related genes under high heme concentration, was shown to improve Hb recombinant titer up to around 7% of the total yeast protein (Martínez et al., 2015). Recently, recombinant Hb yield was reported to reach 18% relative to the total cellular proteins, due to the deletion of *HMX1, VPS10* and *PEP4* involved in heme and mis-folded protein degradation, the overexpression of *AHSP* encoding human α-hemoglobin-stabilizing protein, and the deletion of *ROX1* encoding heme-dependent repressor that inhibits *HEM13* (Ishchuk et al., 2021).

### Cytokines and Growth Factors

Pro-inflammatory (type I) and anti-inflammatory (type II) therapeutic cytokines, such as interferons (IFNs), type I tumor necrosis factors (TNFs) and interleukins (ILs) are known to display a spectrum of activities, including immunomodulatory, antiviral and antiproliferative (e.g., IFN-α treats hepatitis A, B, C and some types of cancer; IFN-β–multiple sclerosis; TNF-α and ILs are employed in anticancer-related therapies) (Lipiäinen et al., 2015; Walsh, 2018; Berraondo et al., 2019). Although recombinant cytokines approved for clinical use are predominantly produced in *E.coli*, several studies were performed to exploit yeasts as expression hosts. For example, the deletion of native lipid regulator gene *OPI1* leads to the endoplasmic reticulum (ER) enlargement and enhanced secretion capacity, which is further emphasized by the overexpression of *CPR5* folding factor and improved protein-folding (de Ruijter et al., 2016). In parallel, to obtain IFN-α 2b in *Y. lipolytica* (Gasmi et al., 2011) and IL-6 in *P. pastoris* (Li et al., 2011), fed-batch cultivation of recombinant protein-expressing strains and purification process were assessed demonstrating recombinant protein high yield and biological activity. Hematopoietic growth factor cytokines, on the other hand, comprise colony-stimulating factors (CSF) including granulocyte colony-stimulating factor (G-CSF) and granulocyte-macrophage colony-stimulating factor (GM-CSF), which are employed as immunostimulants to treat neutropenia (Metcalf, 2010) and are produced since 1991 in *E. coli* (CSF filgrastim) and *S. cerevisiae* (GM-CSF sargramostim) (Table 1).

### Enzymes

Several deficient enzyme disorders (e.g., mucopolysaccharidosis, Pompe disease, Fabry disease, Gaucher disease) and cancers (e.g., lymphoblastic leukemia, melanoma) can be treated by enzyme replacement therapies (Taipa et al., 2019). The majority of the approved recombinant enzymes are produced in CHO cells (β-glucorebrosidase for Gaucher disease, α-galactosidase for Fabry disease, α-mannosidosidase for α-mannosidosis, sulfatases for mucopolysaccharidosis, α-glucosidase for Pompe disease, digestive enzymes), some in *E. coli* (*E. coli* urate oxidase to treat Gout and asparaginase for lymphoblastic leukemia), and *S. cerevisiae* (*S. cerevisiae* urate oxidase for the treatment of hyperuricemia) (Walsh, 2018; Taipa et al., 2019) (Table 1). Nevertheless, the employment of recombinant enzymes remains challenging due to their large size, potential immunogenicity, undesirable side activity, and poor stability. However, the enhancement of enzyme efficiency minimizes the required enzyme concentration, which can be achieved via site-directed mutagenesis/truncation and computational protein design (Yang et al., 2017). Another limiting factor is recombinant enzyme delivery, and several strategies have been investigated to provide a longer serum half-life and targeted distribution *in vivo*, such as fusion proteins, encapsulation (e.g., lysosomes, nanoparticles) and PEGylation (poly[ethylene glycol] attachment), which remain challenging and case-specific (Dean et al., 2016).

## Therapeutic Natural Products

The extensive exploitation of natural resources and prevailing unavailability of chemical synthesis results in recurrent shortages of medicinal NPs, and particularly highly valued plant-derived compounds, such as *Apocynaceae* monoterpene indole alkaloids (MIAs), opium poppy benzylisoquinoline alkaloids (BIAs) and hemp cannabinoids (Cragg and Newman, 2013; Courdavault et al., 2020). Numerous of these compounds, or their precursors, have been biosynthesized in yeast (Table 2), but commonly the titer does not reach industrial scale and requires further improvement. Recently, BIA central intermediate (*S*)-reticuline yielded 4.6 g/L (a spectacular 57,000-fold increase) in engineered *S. cerevisiae*, where eight heterologous enzymes were expressed and the native metabolism was modified to improve the formation and accumulation of 4-hydroxyphenylacetaldehyde (4-HPAA) (Pyne et al., 2020). However, the truncation-optimized norcoclaurine synthase (tNCS) that condensates 4-HPAA and dopamine to form the main precursor norcoclaurine showed cytotoxicity, which was further minimized by targeting tNCS to the peroxisome (Grewal et al., 2021). In parallel, the identification of missing enzymes of the tropane alkaloid (TA) pathway allowed the construction of *S. cerevisiae* strain producing scopolamine (0.03 mg/L) via a combinatorial approach. In addition to the introduction of 15 heterologous and two engineered heterologous enzymes, native arginine and polyamine metabolism were optimized to improve the accumulation of TA precursor putrescine, and endogenous intermediate degradation was addressed (Srinivasan and Smolke, 2019, 2020). On the other hand, *de novo* biosynthesis of highly valuable cannabinoids was reported in *S. cerevisiae*, where nine heterologous genes in tandem with the enhanced native mevalonate pathway and increased geranyl pyrophosphate (GPP) flux resulted in 8 mg/L of tetrahydrocannabinolic acid production (THCA) (Luo et al., 2019). Moreover, another study demonstrated that targeting the entire GPP biosynthetic pathway into peroxisomes minimizes the endogenous competition for GPP and increases up to 125-fold the production of downstream GPP-derived heterologous compounds (Dusséaux et al., 2020). This displays a potential application for further improvement of cannabinoid titer given that *Cannabis sativa* olivetolate geranyltransferase CsPT4, which uses GPP as a co-substrate, is active in the peroxisome. Remarkably, heterologous enzyme promiscuity was assessed in several studies (Srinivasan and Smolke, 2019; Pyne et al., 2020) demonstrating the discovery of new-to-nature compounds with potentially novel biological properties, which is a prodigious feature for drug discovery.

**Table 2:** Plant-derived therapeutic NPs produced in *S. cerevisiae* (adapted and updated from Pyne et al., 2019).

**Compound**	**Plant origin**	**Characteristic(s)**	**Titer (mg/L)**	**Reference**
**Alkaloids**				
MIA				
Strictosidine	*Catharanthus roseus*	Precursor of anticancer MIAs	0.5	Brown et al. (2015)
BIA				
Thebaine;	*Papaver somniferum*	Precursor of therapeutic opioids;	0.006	Galanie et al. (2015)
Hydrocodone		Analgesic	0.0003	
Noscapine	*Papaver somniferum*	Antitussive	2.2	Li et al. (2018)
(*S*)-reticuline	*Papaver somniferum*	Precursor of therapeutic opioids	4,600	Pyne et al. (2020)
TA				
Scopolamine	*Datura stramonium Atropa belladonna*	Neuromuscular disorder treatment	0.03	Srinivasan and Smolke (2020)
**Cannabinoids**				
THCA; CBGA; CBDA	*Cannabis sativa*	Psychoactive THC; Psychoactive CBG; Psychoactive CBD	8 136 0.004	Luo et al. (2019)
**Terpenoids**				
Sesquiterpenoids				
*a*-humulene;	*Zingiber zerumbet*	Anti-inflammatory and	1,300	Zhang et al. (2018)
Zerumbone		anticancer	40	
Amorphadiene	*Artemisia annua*	Precursor of antimalarial artemisinin	37,000	Westfall et al. (2012)
Artemisinic acid	*Artemisia annua*	Precursor of antimalarial artemisinin	25,000	Paddon et al. (2013)
Diterpenoids				
Jolkinol C	*Euphorbiaceae Thymelaceae*	Precursor of therapeutic diterpenoids	800	Wong et al. (2018)
Taxadiene	*Taxus brevifolia*	Precursor of anticancer paclitaxel	129	Nowrouzi et al. (2020)
Triterpenoids				
β-amyrin	Wide distribution	Precursor of therapeutic triterpenoids	279	Liu et al. (2019)
Ginsenoside Rh2	*Panax*	Anticancer	300	Zhuang et al. (2017)
**Phenylpropanoids**				
Stilbenoids				
Resveratrol; Pinostilbene; Pterostilben	Wide distribution	Wide range of beneficial properties	800 5.52 34.93	Li et al. (2016)
Flavonoids				
Naringenin	Wide distribution	Wide range of beneficial properties	>200	Lehka et al. (2017)
Scutellarin	*Erigeron breviscapis*	Cardio/cerebrovascular disease treatment	108	Liu et al. (2018)
Genistein	*Genista tinctoria*	Phytoestrogen, angiogenesis inhibitor	7.7	Trantas et al. (2009)
Pinocembrin;	Wide distribution	Antioxidant, antidiabetic;	2.6	Eichenberger et al. (2017)
Phloretin	Wide distribution	Precursor of hypoglycemic phlorizin	42.7	Rodriguez et al. (2017)
Quercetin		Wide range of beneficial properties	20.38	
Kaempferol	Wide distribution	Wide range of beneficial properties	66.29	Duan et al. (2017)

## Future Perspectives and Challenges

**Figure 1:**
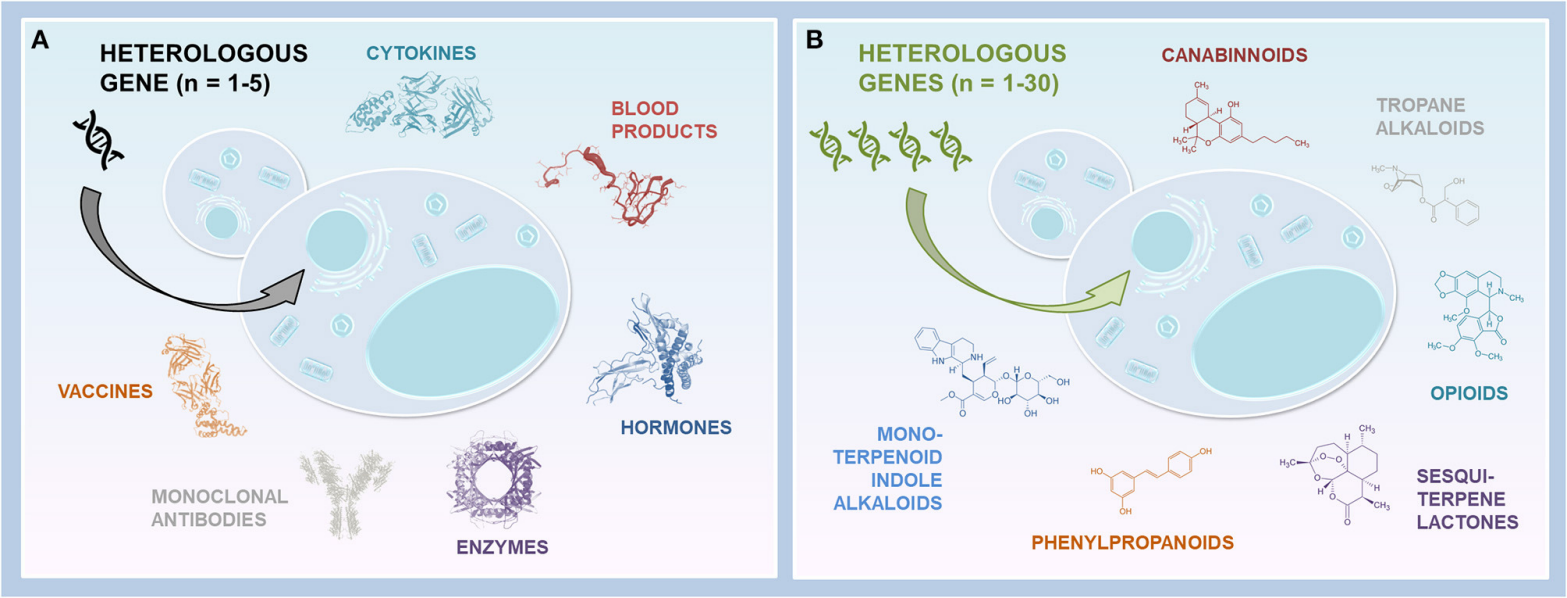
Yeast platforms expressing heterologous genes. **(A)** Yeast cells producing biopharmaceuticals. Few heterologous genes are required to produce recombinant proteins of interest. In some cases, several genes are necessary (e.g., multiple gene copies, fusion proteins, Hb, glycosylation humanization). **(B)** Yeast cells producing NPs. Multiple heterologous genes are introduced into yeast to reconstitute the entire metabolic pathways and produce the compounds of interest, or their precursors.

*E. coli* and yeasts display several advantages compared to cell cultures and transgenic plants/animals in the context of their rapid, cost-effective and easy cultivation, as well as the ease of genomic manipulation. However, given *E. coli* endotoxins and inclusion bodies, as well as the lack of PTM system (discussed in more detail in Tripathi and Shrivastava, 2019), the yeast expression system is frequently emphasized. Thus, although yeast glycosylation differs from human glycosylation, and recombinant protein overexpression may result in insufficient secretion, the development of synthetic biology tools enabled to start addressing these issues (reviewed in Laukens et al., 2015 and Huang et al., 2018). Whereas still challenging, the humanization of yeast glycosylation and improvement of the secretory machinery unveiled the way toward heterologous production of, in principle, any therapeutic recombinant protein, including mAbs (Figure 1A). Moreover, WYVs and yeast potential for the production of recombinant hemoglobin-based oxygen carriers and meat substitutes further spotlight yeast as an auspicious production platform (Lei et al., 2020; Gao et al., 2021; Ishchuk et al., 2021). On the other hand, high-scale heterologous production of therapeutic NPs, particularly plant-derived metabolites, encounters a more extensive range of obstacles due to the complexity of plant metabolic pathways (Figure 1B). Indeed, multi-enzyme pathway subcellular compartmentalization, enzyme promiscuity and poor activity, intermediate cytotoxicity and competing native metabolism require combinatorial strategies. In addition to pathway elucidation, the development and advances of synthetic biology tools, including CRISPR/Cas9 system, fine-tuning of gene expression and enzyme activity, rewiring primary metabolism for cofactor or substrate supply, as well as exploiting subcellular localization (Guirimand et al., 2020), enabled the rise of heterologous pathway reconstitution in yeast. For instance, the optimization of native metabolism is frequently employed to revise metabolic fluxes (Luo et al., 2019; Dusséaux et al., 2020; Pyne et al., 2020; Srinivasan and Smolke, 2020), and enzyme(s) relocalization to the peroxisome appeared as a promising strategy to increase yields (Dusséaux et al., 2020; Grewal et al., 2021). In parallel, with the expanding large high-quality datasets, predictive engineering and systems metabolic engineering emerged as a tool to facilitate the design of desired cell properties via specific genetic modifications (Lee et al., 2012; Zhang et al., 2020). Thus, further implementation of computation and rational strategies in addition to pathway characterization, as well as current and future progress in metabolic engineering, promise a prominent potential of yeast cell factories for any heterologous production, including the new-to-nature compounds.

## Author Contributions

NK, SB, CG, GG, NP, and VC contributed to the writing of this manuscript. All authors contributed to the article and approved the submitted version.

## Funding

We acknowledge funding from the ARD2020 Biopharmaceutical program of the Région Centre Val de Loire (BioPROPHARM, CatharSIS, and ETOPOCentre projects), EU Horizon 2020 research and innovation program (MIAMi project-grant agreement N° 814645), and ANR MIACYC (ANR-20-CE43-0010).

## Conflict of Interest

The authors declare that the research was conducted in the absence of any commercial or financial relationships that could be construed as a potential conflict of interest.

## Publisher's Note

All claims expressed in this article are solely those of the authors and do not necessarily represent those of their affiliated organizations, or those of the publisher, the editors and the reviewers. Any product that may be evaluated in this article, or claim that may be made by its manufacturer, is not guaranteed or endorsed by the publisher.
